# Nuclear export inhibitor leptomycin B induces the appearance of novel forms of human Mdm2 protein

**DOI:** 10.1038/sj.bjc.6600752

**Published:** 2003-02-18

**Authors:** S Menéndez, M Higgins, R G Berkson, C Edling, D P Lane, S Laín

**Affiliations:** 1 Department of Surgery and Molecular Oncology, Ninewells Hospital and Medical School, Dundee DD1 9SY, UK

**Keywords:** leptomycin B, human Mdm2, p53, proteasome, limited proteolysis

## Abstract

The nuclear export inhibitor leptomycin B (LMB) prevents the export of proteins from the nucleus to the cytoplasm, protects p53 from Mdm2-mediated degradation and is a very potent inducer of the p53 transcriptional activity. Here we suggest that LMB can also interfere with the degradation of human Mdm2. In the presence of this drug, we observed two novel forms of this protein: a slow mobility form and an amino-terminal fragment with an apparent molecular mass of 32 kDa. The presence of this 32 kDa band is abolished with proteasome inhibitors, indicating that its appearance could be because of limited processing by the proteasome. These results may be useful in understanding the mechanism of degradation of Mdm2 by the proteasome.

It is well established that approximately 50% of human tumours carry inactivating mutations in the p53 gene (reviewed in Hollstein *et al*, 1999). In many others, the p53 tumour suppressor function is hampered by the overexpression or inactivation of cellular factors that regulate the levels and activity of p53 or by the expression of certain oncoviral proteins (reviewed in Lane and Lain, 2002; Vousden, 2002). Radiotherapy and many of the chemotherapeutic drugs currently used in cancer treatment are potent inducers of the p53 response. Furthermore, a lack of response to these treatments has been frequently associated with mutations in the p53 gene (Pirollo *et al*, 2000; Soussi, 2000). These observations suggest that the activation of p53 is a key determinant for the success of a treatment. However, although effective in many cases, most of the available treatments have a serious disadvantage, namely their DNA-damaging effects, which in the long term may lead to the appearance of secondary tumours. Therefore, the search for novel nongenotoxic activators of the p53 response is widely thought to be essential in improving the treatment of those cancers in which the p53 function is not abolished by mutation.

We have previously shown that the *Streptomyces* sp. metabolite leptomycin B (LMB) induces the transcriptional activity of a p53-dependent reporter plasmid (Lain *et al*, 1999a). This effect of LMB is accompanied by an increase in the levels of the products of two p53-dependent genes, *p21* and *Mdm2* (Freedman and Levine, 1998; Lain *et al*, 1999a) in a p53-dependent manner (Smart *et al*, 1999). As presented here, the increase in the levels of these proteins is accompanied by an increase in the levels of their messenger RNAs (Figure 1A

**Figure 1:**
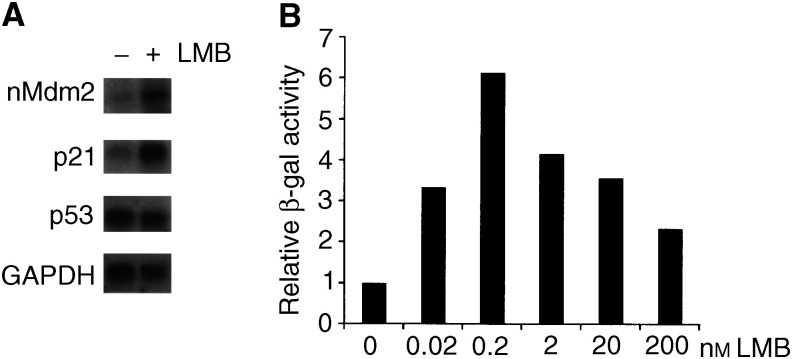
(**A**) LMB increases the levels of Mdm2 and p21 mRNAs. Total RNA was prepared from untreated or LMB-treated human normal primary fibroblasts and the hMdm2, p21 and p53 mRNAs were detected by Northern blotting. GAPDH mRNA was analysed as a control. (**B**) T22 RGCΔfos-lac Z cells were treated with the indicated concentrations of LMB for 18 h and p53-dependent *β*-gal reporter activity was measured.

), firmly establishing that LMB induces the transcriptional activity of p53 and that this occurs without an increase in the levels of p53 mRNA expression (Figure 1B). We also show that the induction of a p53 reporter construct can occur at less than nanomolar concentrations of LMB (Figure 1B), demonstrating the potency of LMB as an inducer of the p53-dependent transcriptional activity.

Accordingly, we have previously reported that LMB has a strong killing effect on cell lines derived from neuroblastomas at nanomolar concentrations in a p53-dependent way, whereas it has only a relatively mild and reversible growth arresting effect on normal human fibroblasts in culture at micromolar concentrations (Smart *et al*, 1999).

In the early reports, LMB was shown to have antitumour effects in mouse xenograft models (Roberts *et al*, 1986) that led to a phase I trial (Newlands *et al*, 1996). Unfortunately, these studies had to be stopped because of the malaise and anorexia induced by the drug when administered systemically by intravenous injection. We then thought that LMB may be of use in the prevention of tumours, such as HPV-associated cervical carcinomas, that can be treated locally and that tend to be recurrent after surgery. This drug increased the stability and the transcriptional activity of p53 in cell lines derived from cervical cancers and effectively induced cell death in a p53-dependent way (Hietanen *et al*, 2000).

These results, together with the potency of LMB in inducing the transcriptional activity of p53, imply that the elucidation of the mechanism of action of LMB is of great importance, and may make it possible to define new ways to target the p53 pathway that yield effective nongenotoxic drugs.

LMB is thought to function primarily as an inhibitor of the export of proteins from the nucleus to the cytoplasm because of its ability to interact with and impair the function of the nuclear export factor crm-1 (Kudo *et al*, 1999). Although other effects of LMB that could lead to a DNA damage response cannot be excluded, LMB is likely to be a very potent inducer of the p53 response, which does not act directly through the DNA damage pathway (Lain *et al*, 1999b).

Middeler *et al* (1997) have elegantly demonstrated that p53 shuttles from the nucleus to the cytoplasm. Additionally, p53 was shown to contain two nuclear export sequences, one in its oligomerisation domain (Stommel *et al*, 1999) and the other in its N-terminus (Zhang and Xiong, 2001), which probably explains its sensitivity to LMB. Direct interaction of p53 with crm-1 has not been reported yet.

In normal nonstressed cells, p53 has a very short half-life because of the following autoregulatory feedback loop mechanism in which the Mdm2 protein plays a key role. It has been well established that wild-type p53 acts as a transcriptional activator of the Mdm2 gene (Barak *et al*, 1993). In turn, Mdm2, which itself has a very short half-life because of its autoubiquitination activity (Fang *et al*, 2000), has the ability to interact with p53 and to function as an E3 ubiquitin ligase that promotes the conjugation of p53 to ubiquitin (Honda *et al*, 1997; Fang *et al*, 2000; Honda and Yasuda, 2000). This conjugation serves as a tag that effectively targets p53 for degradation by the proteasome (Haupt *et al*, 1997; Kubbutat *et al*, 1997). We have previously shown that LMB protects p53 from Mdm2-mediated ubiquitination and degradation (Lain *et al*, 1999a; Xirodimas *et al*, 2001b). Here, we have asked whether LMB has any effects on Mdm2.

## MATERIALS AND METHODS

### Cells and culture conditions

U2OS cells (expressing wild-type p53) and H1299 cells (p53-negative) were obtained from the ATCC. The T22 RGCΔfos-lac Z cell line and the human normal primary fibroblasts (HNF) were described in Lu *et al* (1996) and Lain *et al* (1999a), respectively. Cells were cultured in RPMI (H1299) or DMEM (U2OS, T22Δfos-lac Z and HNF) supplemented with 10% foetal calf serum and gentamycin antibiotic at 37°C in an atmosphere containing 5% CO_2_.

### Chemicals and reagents

Leptomycin B was obtained from Novartis. Actinomycin D was obtained from Sigma. Stock solutions were prepared in absolute ethanol. Proteasome inhibitors (MG132, lactacystin, ALLN, epoxomycin and PI-II) and calpain inhibitors (ALLM and Z-Val-Phe-CHO) were obtained from Calbiochem, and stock solutions were prepared in DMSO.

### Plasmids

pCMVhMdm2 and pCMVhMdm2 mtNLS were a kind gift from Dr A Levine. hMdm2 mtNoLS and the expression vector for p14ARF (pcDNA3p14ARF) were provided by K Vousden. pcDNA3Mdm2 (1–244) and pcDNA3Mdm2(1–258) were described by Midgley *et al* (2000). pCMVhMdm2C462A as well as all other point mutants of hMdm2 were obtained by site-directed mutagenesis. The expression vector for the N-terminal fragment of hMdm2 (pCMVhMdm2(1–244)) was derived from pCMVhMdm2 by insertion of a translation terminator at codon 245. pCOCMdm2×2 was a gift from M Oren. pcDNA3p53 is described in Xirodimas *et al* (2001a). pSVp14ARF was obtained by inserting the *Nco*I–*Xba*I fragment of pcDNA3p14ARF into the *Sac*I and *Xba*I sites of the pSV vector.

### Antibodies

p53 was detected using the DO1 mouse monoclonal antibody (Stephen *et al*, 1995). Human Mdm2 was detected using the 4B2, 3G5, SMP14, 2A9, 2A10 and 4B11 mouse monoclonal antibodies (Chen *et al*, 1993; Picksley *et al*, 1994).

### Northern and *β*-gal reporter assays

Total RNA was obtained from normal human primary fibroblasts and mRNA levels were analysed as described by Hietanen *et al* (2000).

T22RGCΔfos-lac Z cells were seeded at a density of 10^4^ cells per well in 96-well plates. After 24 h recovery time, LMB or control drugs were added and cells were incubated with the drugs for 16 h. Cells were lysed in Promega reporter lysis buffer for 1 h, and then in-cubated with CPRG *β*-gal substrate (Boehringer–Mannheim, Ingelheim, Germany). Colour density was measured at 590 nm using a Dynatech plate reader.

U2OS cells were seeded in 24-well plates and transfected with 60 ng of the RGCΔFos-lacZ p53 reporter plasmid, 60 ng of the control SV-promoter-dependent luciferase reporter and the indicated plasmids expressing hMdm2(1–244) or p14ARF using the Fugene protocol as described by Midgley *et al* (2000). Cells were harvested 48 h after transfection. *β*-gal activity was measured as above and luciferase activity was analysed using the Dual-Luciferase® Reporter Assay System (Promega, Southampton, UK), a MicroLumat LB96 V(EG&G Berthold) luminometer.

### Western blot analysis

Cells were seeded in 10 cm dishes at a density of 9×10^5^ cells per well and transfected using the calcium phosphate method. Equivalent amounts of CMV promoter in the transfections were maintained with the pcDNA3 control vector. The amount of plasmid used in each transfection was topped up to 20 *μ*g with the bacterial plasmid Bluescript. After a 24 h recovery period, 2 nM LMB and the indicated amounts of proteasome inhibitors were added to the cells for 18 h before harvesting. Cell lysates were prepared using the NP40 buffer described by Xirodimas *et al* (2001a) or by direct lysis in SDS–PAGE loading buffer. Equivalent results were obtained with both the methods. Samples were analysed in 4–12% Novex gels using MOPS running buffer after which they were transferred to Immobilon membranes that were incubated with the indicated primary antibodies and developed as described by Xirodimas *et al* (2001a).

### Purification of His-tagged ubiquitin conjugates

For the *in vivo* ubiquitination assay, vectors expressing the relevant proteins were cotransfected with 2 *μ*g of the vector encoding His-tagged ubiquitin. Purification of His_6_-ubiquitinated conjugates was performed as described in Xirodimas *et al* (2001a). His-ubiquitin tagged hMdm2 was analysed by Western blot analysis with 4B2 antibody.

## RESULTS

### LMB induces the appearance of two novel forms of hMdm2

**Figure 2:**
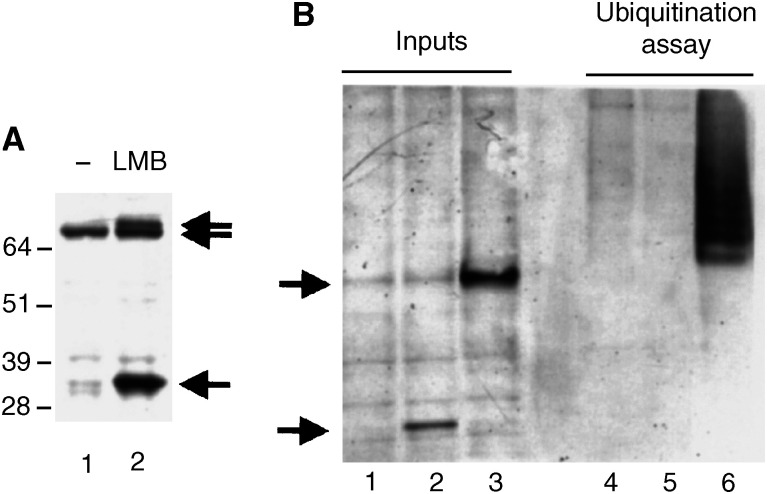
LMB induces the appearance of novel forms of hMdm2. (**A**) H1299 cells were transfected with an expression vector for hMdm2 (pCMVhMdm2) and left untreated (lane 1) or treated with 2 nM LMB for 18 h (lane 2). Cell extracts were analysed by Western blotting and hMdm2 was detected using the 4B2 mouse monoclonal antibody against the amino-terminus of the protein. The positions of the bands corresponding to the full-length hMdm2, the LMB-induced slower mobility form and the 32 kDa fragment are indicated by arrows. (**B**) LMB does not affect hMdm2 ubiquitination *in vivo*. H1299 cells were transfected with 5 *μ*g pCMVhMdm2 and 2 *μ*g pCMVHis_6_–Ubiquitin. In lanes 1 and 3, cells were left untreated, in lanes 2 and 4 they were treated with 2 nM LMB and in lanes 3 and 6, they were also cotransfected with 5 *μ*g of the pcDNA3p14ARF expression vector. In lanes 1–3, total hMdm2 was analysed and in lanes 4–6, His_6_–ubiquitin complexes were isolated and ubiquitinated hMdm2 was detected using 4B2.

Mdm2 has been described to shuttle between the nucleus and the cytoplasm and to contain a crm1-binding nuclear export sequence (Roth *et al*, 1998). Accordingly, Argentini *et al* (2001) found that LMB partly decreases the export of Mdm2 from the nucleus using heterokaryon assays. However, this is not reflected by any convincing changes in the localisation of Mdm2 in the presence of LMB by either immunocytofluorescence or cell fractionation experiments (data not shown). Instead, we observed that LMB induces the appearance of a shorter human Mdm2 (hMdm2) product of approximately 32 kDa, which reacts with the 4B2 mouse monoclonal antibody directed against the amino terminus of the protein (Chen *et al*, 1993). Other insults that are known to activate p53, such as actinomycin D and UVC, did not cause the appearance of this product (not shown). In many experiments, we could also detect that the presence of the 32 kDa band is accompanied by the appearance of another modified form of hMdm2 with a slightly slower electrophoretic mobility than full-length hMdm2 in nontreated cells. As shown in the *in vivo* ubiquitination assay presented in Figure 2B

, unlike proteasome inhibitors and the p14ARF tumour suppressor (Xirodimas *et al*, 2001a), LMB did not increase the levels of ubiquitin-conjugated hMdm2 and the 32 kDa band did not appear to be bound to ubiquitin.

### The appearance of the 32 kDa band in the presence of LMB is suppressed by proteasome inhibitors

**Figure 3:**
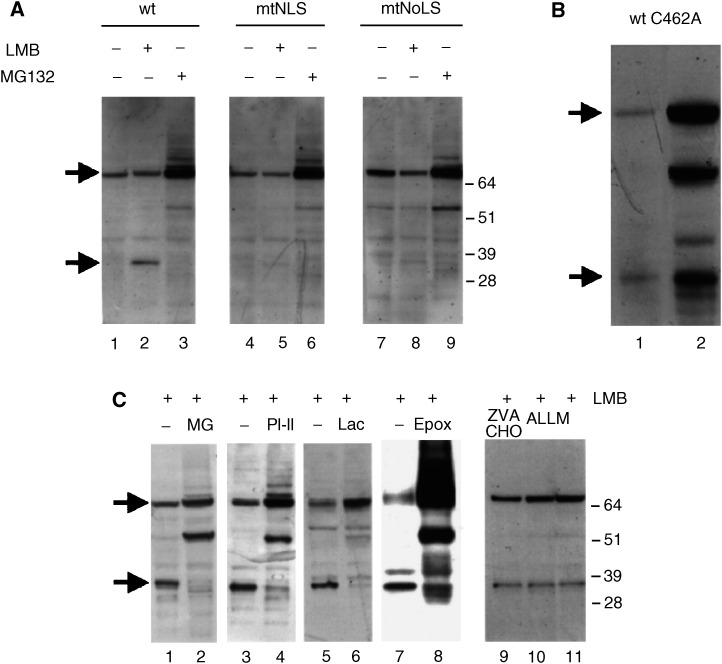
The appearance of the 32 kDa is a post-transcriptional event and is prevented by proteasome inhibitors. (**A**) H1299 cells were transfected with vectors expressing either wild-type hMdm2 lanes (1–3), hMdm2 mtNLS lanes (4–6) or hMdm2mtNoLS lanes (7–9). In lanes 1, 4 and 7, cells were left untreated. In lanes 2, 5 and 8, they were treated with LMB, in lanes 3, 6 and 9, they were treated for 3 h with the proteasome inhibitor MG132 (20 *μ*M). Cells were harvested and hMdm2 was detected by Western blotting as above. The positions of the bands corresponding to the full-length hMdm2 and the 32 kDa fragment are indicated by arrows. (**B**) H1299 cells were transfected with an expression vector for hMdm2 or the hMdm2C462A mutant and treated with 2 nM leptomycin B for 18 h. hMdm2 was detected as above. (**C**) H1299 cells were transfected with 5 *μ*g of the expression vector for hMdm2 (pCMVhMdm2) and treated with 2 nM leptomycin B alone for 18 h (lanes 1, 3, 5, 7 and 11) or together with 20* μ*M MG132 (lane 2), 20* μ*M PI-II (lane 4) or 20* μ*M lactacystin (lane 6), 4* μ*M epoxomycin (lane 8), 10* μ*M Z-Val-Phe-CHO (lane 9) or 10* μ*M ALLM (lane 10). Cell extracts were analysed by Western blotting and hMdm2 was detected using 4B2. The positions of the bands corresponding to the full-length hMdm2 and the 32 kDa fragment are indicated by arrows on the left side.

As shown in Figure 3A

, LMB did not effectively induce the appearance of the 32 kDa band in cells transfected with a human Mdm2 with mutations in its nuclear localisation signal at residues 181–185 (hMdm2 mtNLS) (Tao and Levine, 1999), or in its nucleolar localisation signal (hMdm2 mtNoLS) that is located between residues 466 and 473 (Lohrum *et al*, 2000). This result has two consequences. First, it establishes that the appearance of the 32 kDa band is due to an effect on the Mdm2 protein and not to an effect of LMB on the expression of the transfected vector. Second, it could suggest that the appearance of the 32 kDa band requires that hMdm2 reaches the nuclear and nucleolar comparments efficiently.

In subsequent experiments, we aimed to characterise the activity responsible for the accumulation of the 32 kDa band in the presence of LMB. Mdm2 has been previously described to be susceptible to caspase cleavage, producing a band of approximately 60 kDa (Pochampally *et al*, 1999). However, the levels of the 32 kDa band detected with LMB were not decreased by preincubation with caspase inhibitors (not shown). Instead, we observed that the ratio between the levels of full-length hMdm2 and the 32 kDa product significantly decreased when an hMdm2 variant with a point mutation in its RING finger domain (hMdm2C462A), and therefore deficient for autoubiquitination and degradation (Honda *et al*, 1997), was analysed (Figure 3B). This suggested that the appearance of the 32 kDa fragment could be to some extent dependent on ubiquitination status of Mdm2 and therefore, potentially because of a proteasome-associated proteolytic activity.

As presented in Figure 3C (lanes 2 and 4), the proteasome inhibitors MG132 and PI-II (Z-LeuLeuPhe-CHO) significantly decreased the appearance of the 32 kDa fragment in the presence of LMB. This effect is also observed with the more specific inhibitors of the proteasome activity lactacystin and epoxomycin (Figure 3C, lanes 6 and 8), but not with the calpain inhibitors Z-Val-Phe-CHO and ALLM (Figure 3C, lanes 9 and 10). These results suggest that the appearance of the 32 kDa fragment may depend on the activity of the proteasome. The band that appears below the 64 kDa marker after long-term incubation with proteasome inhibitors (Figure 3C) and with the hMdm2C462A mutant (Figure 3B) could be related to the caspase-dependent cleavage (Pochampally *et al*, 1999).

**Figure 4:**
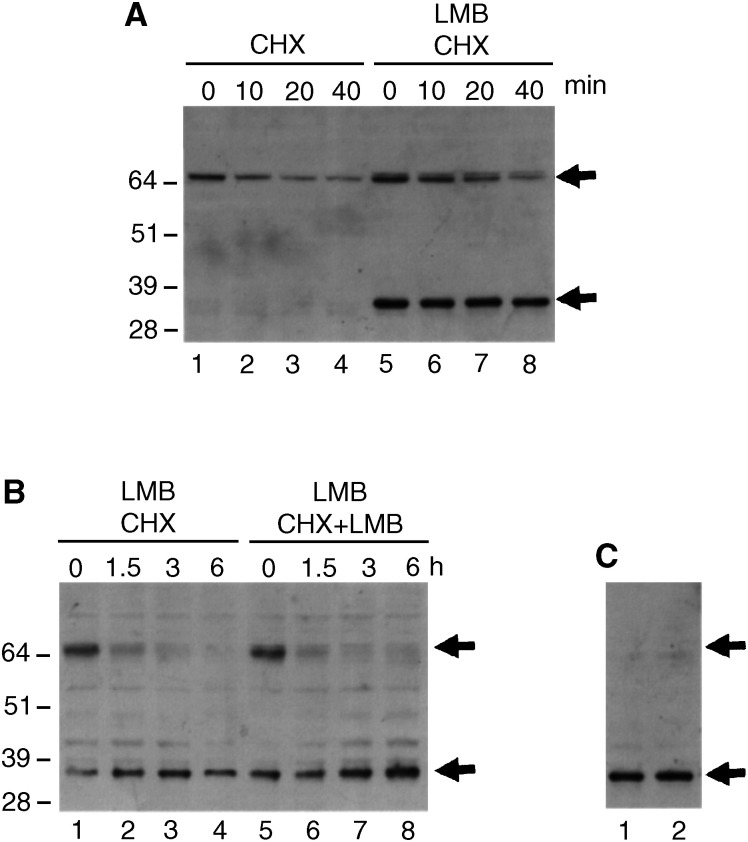
Analysis of the stability of hMdm2 in the presence of LMB. (**A**) H1299 cells were transfected with the expression vector for hMdm2 and left untreated (lanes 1–4) or pretreated with 2 nM LMB for 18 h (lanes 5–8). After this time, 10* μ*g ml^−1^ cyclohexamide (CHX) was added to the medium and the cells were harvested after 0, 10, 20 and 40 min. Cell extracts were analysed by Western blotting and hMdm2 was detected using 4B2. The positions of the bands corresponding to the full-length hMdm2 and the 32 kDa fragment are indicated by arrows. (**B**) H1299 cells were transfected with the expression vector for hMdm2 and pretreated with 2 nM LMB for 18 h. After this time, the medium was removed and replaced with a medium containing 10* μ*g ml^−1^ CHX (lanes 1–4) or a medium containing 10* μ*g ml^−1^ CHX and 2 nM LMB (lanes 5–8). Cells were harvested after 0, 1.5, 3 and 6 h and cell extracts were analysed by Western blotting and hMdm2 was detected using 4B2. (**C**) H1299 cells were transfected with the expression vector for hMdm2 and pretreated with 2 nM LMB for 18 h after which 10* μ*g ml^−1^ CHX was added for 4 h. At this time, virtually all detected hMdm2 has an apparent molecular weight of 32 kDa. In order to see whether removal of LMB destabilised the 32 kDa fragment, we removed the medium and replaced it with a medium containing 10* μ*g ml^−1^ CHX only (lane 1) or a medium containing 10* μ*g ml^−1^ CHX and 2 nM LMB (lane 2). After 5 more h, cells were harvested and analysed as above.

**Figure 5:**
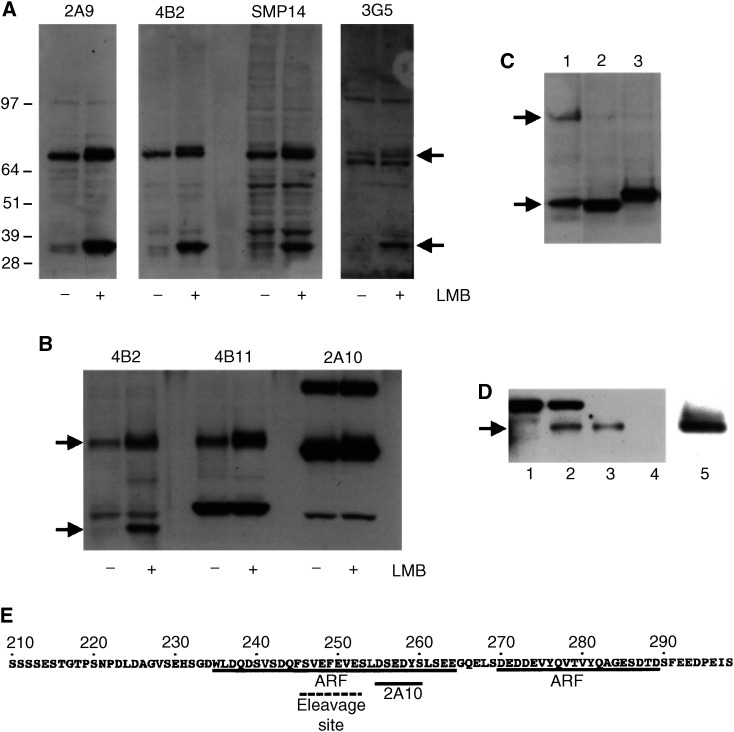
Mapping of the 32 kDa band C-terminal region. (**A** and **B**) H1299 cells were transfected with an expression vector for hMdm2 (pCMVhMdm2) and left untreated (−) or treated with 2 nM LMB for 18 h (+). Cell extracts were analysed by Western blotting and hMdm2 was detected using the mouse monoclonal antibodies 2A9, 4B2, SMP14 and 2A9 (**A**), or 4B2, 4B11 and 2A10. (**B**). The positions of the bands corresponding to the full-length hMdm2 and the 32 kDa fragment are indicated by arrows. (**C**) H1299 cells were transfected with an expression vector for hMdm2 (pCMVhMdm2) (lane1), a vector expressing residues 1–244 of murine Mdm2 (pcDNA3Mdm2 1–244) or a vector expressing residues 1–258 (pcDNA3Mdm2 1–258) (lane3). pcDNA3Mdm2 1–244 and pcDNA3Mdm2 1–258 contain six and 14 additional residues at their C-terminus, respectively. (**D**) H1299 cells were transfected with expression vectors for hMdm2(1–252N_47_) (lanes 1 and 2) or hMdm2 (lanes 3 and 4). In lanes 2 and 3, cells were treated with 2 nM LMB for 18 h. Cell extracts were analysed by Western blotting and developed with the 4B2 antibody. The position of the 32 kDa band is indicated with an arrow. In lane 5, cells were transfected with the plasmid encoding the hMdm2 (1–244) mutant. This fragment is expressed at very high levels, and therefore, a shorter exposure of the gel is shown for this lane. (**E**) Sequence of the acidic domain in hMdm2. The regions involved in the interaction with the amino-terminus of p14ARF according to Bothner *et al* (2001) and the 2A10 epitope are underlined. The region proposed to contain the C-terminus of the 32 kDa band is marked with a discontinuous line.

The cleavage did not seem to be because of an induction of a proteolytic activity, since the presence of the 32 kDa fragment was never accompanied by a decrease in the levels of full-length hMdm2 (Figures 2A, B, 3A and 5A, B). This suggested that LMB could increase the stability of this fragment. However, we observed that the 32 kDa band is very stable (Figure 4A, B

), and that it was not significantly destabilised after removal of LMB from the medium (Figure 4C).

### Mapping of the cleavage site generating the N-terminal 32 kDa fragment

As shown in Figure 5A

, the 32 kDa fragment detected in the presence of LMB contains epitopes 4B2, 3G5, SMP14 and 2A9 reacting with the amino-terminal half of Mdm2 (Chen *et al*, 1993; Picksley *et al*, 1994). Instead, the 2A10 and 4B11 antibodies, that react with the C-terminus of Mdm2 (Chen *et al*, 1993) do not recognise the 32 kDa band (Figure 5B). We have not been able to find the C-terminal fragment resulting from this cleavage with any of these two antibodies. Several additional bands were obtained with the C-terminal antibodies 2A10 and 4B11 (Figure 5B); however, these bands also appear in cells not overexpressing hMdm2 (not shown) and independent of the presence of LMB (Figure 5B).

Using a series of deletion mutants of murine Mdm2 (Midgley *et al*, 2000), we were able to roughly map the region containing the C-terminus of this 32 kDa N-terminal fragment of Mdm2 (Figure 5C). According to this measurement, the C-terminus of this fragment is located approximately within amino 245 and 272 of human Mdm2. The 2A10 antibody has been shown to recognise two epitopes in Mdm2: one between positions 255 and 260, and a C-terminal epitope at positions 388 and 392. The lack of 2A10 reactivity of the 32 kDa fragment allowed us to map the C-terminus of the 32 kDa fragment more accurately to a region between residues 245 to approximately 260. Interestingly, this region is contained within a a region that is bound by the p14ARF tumour suppressor (Midgley *et al*, 2000; Bothner *et al*, 2001) (Figure 5E). However, we have not been able to show that p14ARF overexpression affects the levels of the 32 kDa LMB-induced band (not shown).

We then proceeded to substitute the amino acids in this region by alanine using site-directed mutagenesis. Residues 245, 246, 247, 248, 249, 250, 251, 252, 253, 254, 255, 256, 259, 260 and 261 were substituted. Despite our efforts, we have not been able to identify a specific residue required for this cleavage event. However, a fortuitous mutation allowed us to map the cleavage site more accurately. When introducing a point mutation at position 254, an open reading frame encoding a frame shift mutant of hMdm2 was obtained. This mutant finished at position 252 of the hMdm2 sequence and had 47 extra residues at its C-terminus encoded by an alternative reading frame (hMdm2(1–252N_47_)). When cells expressing this mutant were treated with LMB, the 32 kDa band still appeared (Figure 5D). This indicates that the C-terminus of the 32 kDa band is before or at position 252.

### An N-terminal fragment consisting of residues 1–244 from hMdm2 can protect p53 from degradation by Mdm2

**Figure 6:**
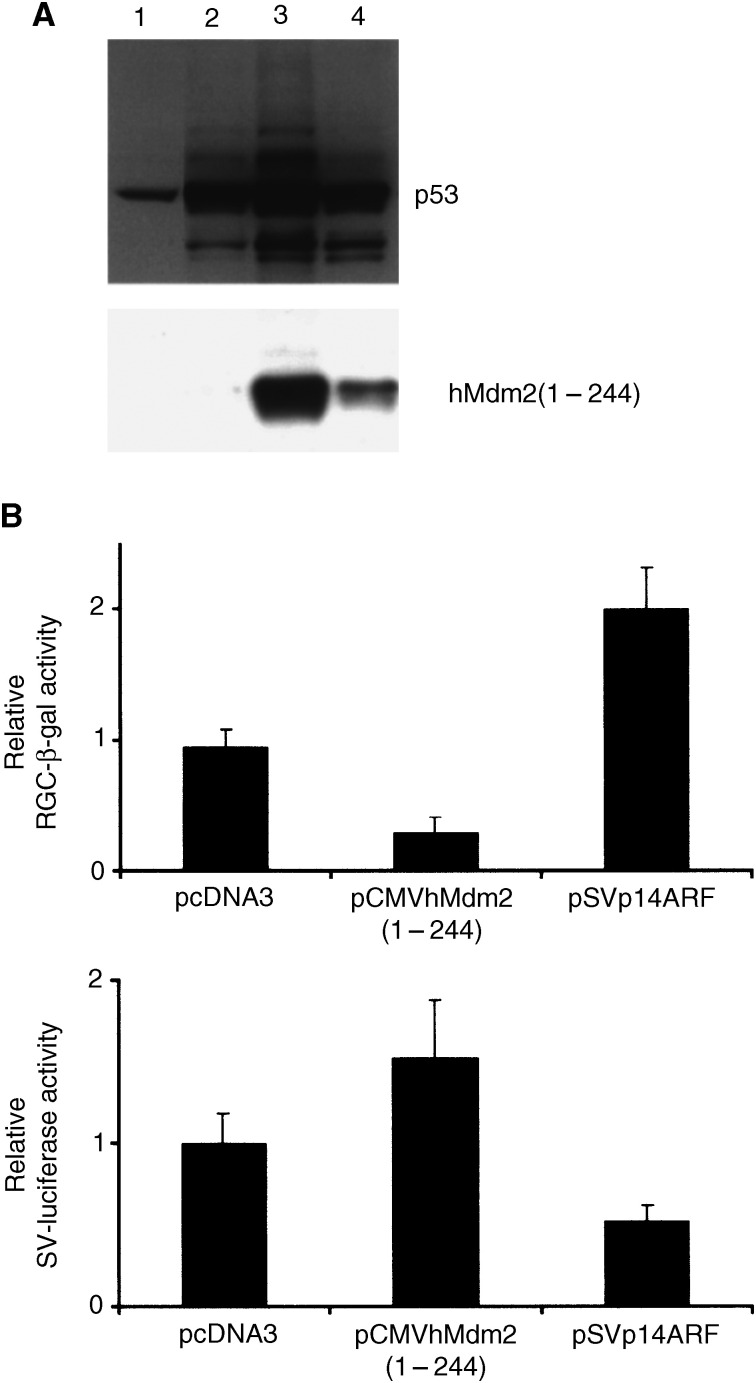
The 1–244 fragment of hMdm2 protects p53 from Mdm2-mediated degradation, but does not increase the transcriptional activity of p53. (**A**) H1299 cells were transfected as described with 2 *μ*g of pcDNA3p53 and 2 *μ*g of pCOCMdm2×2 (lane 1), 2 *μ*g of pcDNA3p53 (lane 2), 2 *μ*g of pcDNA3p53, 2 *μ*g of pCOCMdm2×2 and 15 *μ*g of pCMVhMdm2 (1–244) (lane 3), or 2 *μ*g of pcDNA3p53, 2 *μ*g of pCOCMdm2×2 and 7.5 *μ*g of pCMVhMdm2 (1–244) 1 *μ*g (lane 4). Cells were analysed by Western blotting using the DO1 antibody against p53 (upper panel). hMdm2 (1–244) was detected using the 4B2 antibody against Mdm2 (lower panel). (**B**) U2OS cells, which contain endogenous wild-type p53, were transfected with the p53-dependent RGC*Δ*Fos-lacZ and the SV-luciferase reporter plasmids together with either 0.25 *μ*g pcDNA3 control vector, 0.25 *μ*g pCMVhMdm2 (1–244) or 0.025 *μ*g pSVp14ARF. p53-dependent *β*-gal and control SV40 early promoter-driven luciferase reporter activities were measured. The average and the s.d. of four transfections with each set of constructs is shown.

To study the significance of this 32 kDa fragment for p53 stabilisation and activity, we created an expression vector for an hMdm2 deletion mutant that contains the first 244 amino acids from the N-terminus (pCMVhMdm2(1–244)) (Figure 5D, lane 5). This deletion mutant was able to protect p53 from degradation by full-length murine Mdm2 (Figure 6A

), possibly by competing with Mdm2 for its interaction with p53. This fragment of hMdm2 contains the region necessary for binding to p53 and binding of Mdm2 to p53 is thought to be sufficient to inactivate the transcriptional activity of p53 (Chen *et al*, 1996). Therefore, as could be expected, this deletion mutant could not induce the transcriptional activity of p53 (Figure 6B). This lack of activation was not because of a toxic effect of the pCMVhMdm2 (1–244) vector as indicated by the analysis of the control luciferase reporter activity, where hMdm2 (1–244) expression significantly increases this activity.

## DISCUSSION

We have shown that the electrophoretic behaviour of hMdm2 is altered significantly in the presence of the nuclear export inhibitor LMB. LMB induces the appearance of two novel hMdm2 products, namely a band migrating slightly slower than the major hMdm2 band in the absence of drug, and a product with an apparent molecular weight of 32 kDa, containing epitopes from the N-terminal part of hMdm2. The C-terminus of this fragment maps between residues 245 and 252 of hMdm2.

The 32 kDa N-terminal fragment of hMdm2 may have some relevance for the observed stabilisation of p53 by LMB as evidenced by the ability of transfected truncated hMdm2 to protect p53 from Mdm2-mediated degradation, possibly by acting as a dominant negative towards Mdm2. However, although LMB protects p53 from murine Mdm2-mediated ubiquitination and degradation (Xirodimas *et al*, 2001b), the corresponding 32 kDa form of murine Mdm2 has never been detected. Therefore, although the accumulation of the 32 kDa fragment may contribute to the stabilisation of p53 by LMB, it is not indispensable. Additionally, the induction of the accumulation of the 32 kDa cleavage product of hMdm2 is not likely to contribute to the potent activation of p53-dependent transcription by LMB, since a similar fragment does not increase, but rather decreases, p53 activity in a transcriptional reporter system. One possibility is that the accumulation of the 32 kDa fragment negatively modulates the p53 transcriptional activity and contributes to diminishing the cytotoxic effects of LMB.

The appearance of the 32 kDa fragment occurs without significant decrease in the level of full-length hMdm2. However, this does not seem to be because of alterations in the expression of ectopic hMdm2 cDNA, since different small mutations at distant sites of the protein (the NLS and the NoLS) prevent the detection of the 32 kDa band. The levels of the 32 kDa band did not seem to be related to the nuclear export signal in hMdm2 proposed by Roth *et al* (1998), since this band also appeared when cells transfected with a mutant for this putative nuclear export signal were treated with LMB (not shown).

Inhibition of crm1-mediated nuclear export by LMB could alter some event in the degradation of hMdm2 by the proteasome, allowing an amino-terminal product to accumulate when it would not otherwise do so. Supporting this hypothesis, we have shown that the appearance of the 32 kDa band is clearly prevented by several proteasome inhibitors. Although it is possible that proteasome inhibitors, and even lactacystin, may be affecting other proteolytic activities in addition to that of the proteasome (Ostrowska *et al*, 2000), given the diversity of proteasome inhibitors having the same effect, it is unlikely that the appearance of the 32 kDa fragment is not because of limited proteolysis by the proteasome.

We have shown that the 32 kDa band corresponds to the amino-terminal half of the protein and we have determined that the C-terminus of the 32 kDa band lies within residues 245 to approximately 252 of the hMdm2 sequence. However, extensive point mutagenesis of this region did not abolish the proteolytic event. It is interesting to note that in the case of the NF*κ*B p105 precursor, which is known to be susceptible to partial proteasome-mediated proteolysis to generate the p50 active fragment (Palombella *et al*, 1994), it has not been possible to precisely map the cleavage site. This indicates that the cleavage of hMdm2 and p105 are not sequence dependent, but may rely on recognition of a structural feature. In the case of *N*F*κB*, a glycine-rich region (GRR) between positions 375 and 401 has been shown to be involved in the generation of the p50 form that is thought to occur at approximately position 433 (Lin and Ghosh, 1996). No similar GRR can be found in the hMdm2 sequence.

The cleavage site that would generate the 32 kDa band maps to a conserved and unstructured region in hMdm2 rich in acidic residues and serines (Bothner *et al*, 2001). There is also data showing that this acidic domain is important for the stability of Mdm2. As reported by Argentini *et al* (2001), deletion of the acidic domain (residues 222–272) increases the stability of the protein without decreasing its ubiquitination. Additionally, the interaction site for the amino terminus of p14ARF, which is necessary for p14ARF to increase the levels of Mdm2 and its ubiquitinated forms (Xirodimas *et al*, 2001a), is also in this acidic region (Midgley *et al*, 2000; Bothner *et al*, 2001). PEST sequences are conformationally flexible regions that do not show a high conservation of exact sequence, but contain a high proportion of proline (P), glutamate (E), serine (S) and threonine (T). This type of sequence is considered to target proteins for degradation by the proteasome and possibly other proteolytic systems (Rechsteiner and Rogers, 1996). The importance of the acidic domain for Mdm2 stability and the data presented here could suggest that the acidic region of Mdm2 may act as a PEST sequence.

The C-terminal fragment generated from the limited proteolysis of the p105 precursor of *N*F*κB* has not been detected and there is a lack of precursor–product relation between the p105 and p50 forms (Lin *et al*, 1998,^2000^). This behaviour is also observed here for full-length hMdm2 and the 32 kDa fragment. In the case of *N*F*κB*, these phenomena have been proposed by some authors to be because of cotranslational processing of the protein during synthesis (Lin *et al*, 1998,^2000^). This model is unlikely to be compatible with our observations since mutations near the C-terminus of the protein (at the NoLS) impair the appearance of the 32 kDa band.

Another way to explain our observations is that in normal conditions, hMdm2 undergoes a first proteasome-dependent cleavage within the acidic domain and that in the presence of the nuclear export inhibitor LMB, the N-terminal portion persists, while the C-terminus is degraded. This lack of further degradation of the N-terminal region when nuclear export is inhibited could be because of a direct effect of inhibition of nuclear export on the 32 kDa fragment, which retains its nuclear export signal at positions 197–211 (Roth *et al*, 1998). However, we have not been able to see that the 32 kDa fragment specifically accumulates in the nuclear compartment (unpublished data) and as mentioned before, the nuclear export mutant of hMdm2 behaves exactly as the wild-type. LMB could also have an effect on the activity or localisation of proteasomes. This is unlikely since we have shown that the full-length hMdm2 is still effectively degraded in LMB-treated cells and that the 32 kDa fragment is very stable and does not seem to be further stabilised by LMB.

Alternatively, inhibition of nuclear export by LMB could induce a modification at the amino-terminal half of the full-length protein and this modification could prevent the amino 32 kDa fragment of hMdm2 from complete degradation. This ‘protective’ modification may be related to the appearance of the slower migrating form of full-length hMdm2 with LMB. Interestingly, the PI3-kinase inhibitor LY294002 induces the accumulation of full-length hMdm2 and an apparently equivalent N-terminal 32 kDa fragment of hMdm2 to that seen with LMB (Menendez S, unpublished data).

In summary, the observations presented here on the effect of LMB on hMdm2 indicate that the degradation of hMdm2 could be a stepwise process and therefore it can be limited in particular circumstances.
